# Second Annual Meeting of the American Dental Convention

**Published:** 1856-10

**Authors:** Wm. Henry Burr


THIRD DAY.—MORNING SESSION.

The Convention met at 9 o'clock.

Dr. TAFT moved to close the discussion on "The best preparation of gold for filling teeth," at twelve o'clock, which was carried.

Dr. DIXON, of Pottsville, remarked that the impression left on his mind by the discussion last evening, was this: That those who had been in the habit for years of using gold foil could make a better filling with it than they could with crystalline gold, while those who had used the crystalline gold to a great extent could make the best fillings with that. He objected to the shifting from one substance to another by the profession at large, in order to arrive at conclusions as to the best preparation for filling teeth. Was it not better to leave the experimenting to those who had turned their attention exclusively to some particular preparation. He must confess that, so far as the use of crystalline gold was concerned, he had a very limited experience. He preferred foil, but thought any dentist could do best by using the preparation to which he was most accustomed. He had seen gold foil fillings which could not be excelled, and instanced one that came under his notice, done by Dr. Townsend. It became necessary to take it out, and he found it as hard as amalgam. It was difficult to prevent fillings from becoming moist, but a good filling could be made with foil, even if it does become a little damp.

Dr. W. B. ROBERTS, of New York, said that there were radicals in the dental profession, as well as in politics. He had tried everything for filling, and he had found that in some cases, sponge or crystalline gold could be used where foil could not be used as well, while in other cases foil was best adapted. He practiced on the eclectic principle in this respect.

Dr. C. A. KINGSBURY, of Mt. Holly, N. J., knew of only two preparations of gold for filling teeth, in the form of foil and sponge gold. The question ought more properly to read, which was the best of the two? Both preparations were good. He had been using gold in the form of foil for seventeen years. The crystal gold used at present was very different from the sponge gold first used by the profession. He had seen many cases of operations by dentists who had filled teeth with sponge gold, and he found that a large number of them had proved an entire failure. After being in the teeth a few months, he had found this sponge gold in a very porous and disintegrated state, and he had often found it necessary in these cases, for the preservation of the teeth, to remove the fillings and refill them with gold foil. Most excellent fillings could be made, however, with the crystal gold now used. He had used over an ounce of it making some fillings that gave him satisfaction. He thought there was a disposition to ascribe too much to one article to the exclusion of the other; it was yet to be decided, he thought, which was the best of the two.

He differed from Dr. Dixon in relation to experimenting. In the language of Liebig, nature speaks to us in a peculiar language; she answers at all times the questions we put to her, and such questions are experiments. An experiment is the expression of a thought; we are near the truth when the phenomena elicited by the experiment are corresponding to it, and when the reverse is the result, we may take it for granted that the question is falsely stated and the conception founded in error. He believed thorough experimenting was the only course to arrive at truth.

Dr. Dixon said that in speaking of experiment, he meant to refer to that flood of experiment which some seemed disposed to indulge in. He was glad to see these experiments made in a proper way, and hoped they would be continued.

Dr. T. L. Buckingham, of Philadelphia, suggested that he would like to hear some one tell how to use crystalline gold. There were annealed gold, cylinders, pellets, ropes, and ribbons, each requiring a peculiar manipulation. They had been told that sponge gold was the very best article, but no one had given them a description of the manner in which to use it. He must confess that he had failed, in most cases, in attempting to use it

It was said that it could be welded together and made perfectly solid. What was meant by the term solid? It was applied to metals to indicate a peculiar cohesive, crystalline structure. It was no evidence because you had the largest amount of matter in a given bulk that the particles were cohesive. Ice occupies a larger space than water, but is much more cohesive? We know nothing about the nature of cohesive attraction except the effects produced. Certain metals in a pure state were capable of being welded without heat. Could they weld particles of gold so as to become perfectly hard and cohesive? Wet clay could be compressed so as to have more material in the same bulk than was contained in a burnt brick, and yet the clay was held together by mechanical adhesion, and the brick by cohesive attraction. This was mere theory, but it deserved attention, for in applying this sponge gold in his own practice, though the fillings appeared to be solid, they had broken down and washed away like clay. Were not the particles therefore held together by the same force as pressed clay? Did it not require vitrefaction or fusion to make the gold solid? The process of annealing was done by means of a high temperature; might not the heat cause a change in the arrangement of the particles by which the attraction of cohesion was increased? Even at a low temperature, gold by rolling was rendered stiff and hard; heated in the fire and it became soft again. His own opinion was that they could not weld crystalline gold by the process of filling. He had seen fillings of this material where the surface was not burnished, absorb repeatedly drops of moisture. He believed he had not been deceived in that experiment. If that was so, would not moisture penetrate the whole lump and break it up? It was possible that it might be held together by the interlocking of the crystalline particles of gold, the same as gold foil when packed into a cavity.

Dr. C. S. Weeks, of Bedford, N. Y., had used crystalline gold but little. His first attempt three years ago with sponge gold was a total failure. About a year since he began to use the new crystalline gold, and after several unsuccessful attempts he at last succeeded in some kinds of cavities. He found great difficulty in keeping some cavities dry, but where they were shallow and easily accessible, he could make a better filling than with foil, while with deep cavities not easily got at, he could succeed better with foil.

Dr. AUSTIN, of Baltimore, said that if a crystalline gold filling when subjected to the test was malleable and ductile, it was therefore held together by an equally powerful force with that called cohesion, and it mattered not whether it was actual cohesive attraction or not. If crystal gold could be made as compact as coin it was a new discovery. He did not regard the illustration of the burnt brick and compressed clay as hardly in point. Before going into the fire the elementary substances were in a state very different from what they were on coming out; the silica and alumina became a new chemical compound which possessed cohesive attraction. Reduce that brick to powder, and it was no longer cohesive, or capable of as much cohesion as the clay—in fact, it was not clay, it was brick dust. If a plug of crystalline gold could be rolled out into a thin plate, and drawn into wire, and having the same specific gravity, it followed that it was as solid as coin. Because metals needed annealing, it did not follow that they were not as solid as before. There was some mysterious agency in heat, but he could not see how a crystal gold plug differed essentially from molten gold, if the specific gravity was the same.

Dr. A. MERITT ASSAY, of Philadelphia, gave an account of some experiments made by him, corroborating the remarks of the last speaker, and showing that crystalized gold became solidified in the cavity. He had tested a filling made with sponge gold by the hammer and rollers making it into a very thin plate. He happened to have that specimen in his pocket-book, which he exhibited to the Convention. He had used probably some six ounces of sponge gold and had yet to see the first discoloration of a tooth. He had used it in cavities where without it he was sure he would have been obliged either to use amalgam or extract the tooth. He intended to use it more freely hereafter. No more difficulty attended the use of it than in the case of gold foil. Great care in either case was required to keep the cavity dry; a wet filling of sponge gold would in time peel off or crumble just as a wet filling of foil would do.

Instead of using the sponge gold in little round pieces, he thought it should be applied in flat pieces and packed with an instrument much finer than those made by the manufacturers. He ground his instruments off and serrated them to suit himself.

Dr. WATT, of Ohio, rose to correct the statement of Dr. Buckingham about the impossibility of welding gold except by heat. Gold was one of the welding metals without heat, as every worker in it knew.

Dr. RICH stated that not only gold, but tin and lead, were weldable when cold.

Dr. GEORGE C. WHITE, of New York, said it was but recently that their attention had been called to crystal gold for filling. They knew, on the other hand, that foil had been used from the beginning of dentistry. They had seen what others had done in the use of foil—that fillings had been inserted in the teeth, and had preserved them for generations. On the other hand, fillings had been introduced by other persons, which it had been necessary to renew from year to year, until finally the teeth themselves were destroyed. Two points were necessary to be reached in filling the teeth : first, thoroughness of operation ; and second, skilfulness of manipulation. With these two, any honest man could succeed, either with foil or crystal gold.

Dr. CLARK, of New Orleans, thought that if foil was properly used, and its properties were correctly understood, it would accomplish what every honest dentist would desire to accomplish, the preservation of the teeth. He would undertake to build up a five-cent piece into the shape of a thimble by gold foil of even layers and straight, smooth laminæ, with the pressure of five pounds only, applied with a single point, and any gentleman here could do the same. Still he was much interested in sponge gold, thinking it might be a valuable adjunct to foil. They could do wonderful things with foil, but there seem to be properties in sponge gold not possessed by any other material. He could not say that he could do everything with foil that could be done with sponge gold. There were certain properties about the use of well prepared crystal gold that led him to believe that there were cases which could be treated with more facility by its use than gold foil. He related an achievement of Dr. Allport, of

Chicago, in restoring the exterior and cutting edge of teeth, which to him was more gratifying to look upon than the productions of a Raphael. The front incisors were separated as if a file had been passed between them a quarter of an inch thick, nearly down to the gum. These teeth had been built up and restored to their original shape, their approximate edges almost touched, and they were perfectly adapted to mastication. They had been used nineteen months. He understood that Dr. Allport used foil in connection with crystalline gold in the same cavities. He intended when he went home to try the article.

Dr. Allport having been solicited to state how he proceeded to fill teeth in such cases as these, said that he generally used crystal gold entirely; yet in many difficult cases, for instance, in building up a tooth where two sides were standing, he used more cylinder than crystal gold. He conceived that foil could not be used in particular cases as successfully as crystal. Miracles almost had been performed with gold foil. He had seen fillings made by Dr. Blakesly, of Utica, thirty-five years ago, as perfect now as then, so far as the preservation of the teeth was concerned. One great desideratum in all operations was the saving of time; in this respect he did not regard crystals as advantageous as foil. In many cases he could save one-third or one-half the time with foil. He used also pellets for cases where they seemed better adapted. In this respect every one must use his own judgment; the great thing in filling teeth was the exercise of good sterling common sense. It required more time to learn to use crystal gold, in his judgment, than foil. It required months of hard labor to learn to fill a tooth with gold foil properly, and it required more to learn to do it with crystal gold. But when the art of using it was once acquired, more could be accomplished with it than with foil.

It was said that crystal gold fillings would break down, and that the bottom of the cavities would become soft. Last January he had filled a superior cannine tooth with crystal gold; the whole of the bone of the tooth was gone, there being nothing left but the enamel, and that so thin, that when excavating the cavity the instrument could be seen through it. This filling was worn until April, when the crown was broken off, by biting upon something hard, and came away in pieces, leaving some fragments of the tooth remaining. He saw that tooth two or three days ago, when he filed the edges where the filling came in contact with the enamel, and polished them. There that plug remained to-day, perfect for all the purposes of mastication. He cited this to show that crystal gold would not crumble, if it even did, it was owing not so much to the gold as to the manner of using it. And here he would remark, that much of Dr. Watt's gold made previous to the last year was bad, and even now he regretted to say, much of it was not what could be desired; though it was said to be all alike, there was a difference from some cause or other. The difference was in the working properties, some would adhere almost instantly, while other portions would not.

Dr. SEABURY used more foil than crystalline gold, but in certain cases he knew he could accomplish what he could not with foil. Dr. Allport expressed his experience.

Dr. ARTHUR, of Philadelphia, had in various times and places borne testimony to sponge gold, and had not yet ever any reason to change his opinion. Any failures in his operations he could attribute to some defect of manipulation or some other circumstance which made it exceedingly difficult to perform a good operation. Though some of the fillings were after a lapse of time, not perfectly satisfactory in appearance, yet even then, there was no discoloration below the surface, and no diminution of the density of the filling.

But he no longer used sponge gold; its use had led him to the discovery of an entirely different method of operating from what he had previously pursued, and he had since used gold foil in an entirely different manner. He had been told by a number of gentlemen that this was no new thing, that they had been in the habit of depending upon the adhesive quality of gold foil for years. Such had not been the case with him and many others that he knew. A great many manufacturers of gold foil had been lead to make an effort to avoid the objection of the want of an adhesive quality, which prevented making a good operation when used in the ordinary way. But he was assured by gentlemen who had worked in gold foil, that it was only necessary to produce a perfectly pure article of gold, to possess without annealing this strong adhesive quality, so that it could be welded together in a solid mass like sponge gold. If therefore gold foil could be made to adhere in the same manner as sponge gold, why could it not be used in the same manner and with the same results? Every manufacturer, he had been told, could make it with this adhesive quality, and it could be used precisely as sponge gold, with the exception that the instrument should be somewhat sharply serrated and somewhat more hardened. It was well known that gold foil, no matter how adhesive in the beginning, in a very short time, if exposed to the atmosphere, would lose this adhesive property. But this change which was confined entirely to the surface, could be entirely removed, and the gold restored to its original condition by subjecting it to a very moderate heat—something short of a red heat—ordinarily called annealing. It was only necessary to place it upon a plate and hold it over a spirit lamp until the plate becomes hot. In an article published by a prominent writer on electric metallurgy, it was stated that any metal held in a current of air becomes covered with a film of air, and that it is impossible to get a galvanic deposit upon that surface until it is exposed to heat. It could not be a film of moisture because a sheet of paper could be passed into water and removed perfectly dry. He had endeavored to call the attention of the profession to this method of using gold foil, and a number about Philadelphia were now using it to the exclusion of every other, and said that no inducement could bring them back to any other material. Dr. A. referred to an operation performed by Dr. Colwell, where foil was used in an upper molar tooth, with nothing remaining but the interior surface and a small portion of the outer wall, the most beautiful operation he ever saw, which could not be surpassed by the use of crystal gold.

Dr. Miller, of Massachusetts, said that he had some experience in the use of crystal gold, and he regarded it as a very valuable auxiliary to the profession. He had no doubt that this form of gold did possess valuable qualities, for upon that subject, they had been enlightened with the discoveries of men of experience and learning. He had used sponge gold and cylinders in combination, and they worked well together under certain circumstances. He was an eclectic himself, and used the best material at his command adapted to suit the particular case in hand. When a new thing was introduced into the profession he investigated its merits, and adopted whatever he found to be valuable about it. He knew of no other way to perfect success. Life was made up of experiments. If objection was made to experimenting, it would cut off all improvement. His object in the practice of his profession was, to make a thorough examination of all new discoveries in the line of his art, and adopt whatever was practical and valuable.

Dr. SEARLE, of Springfield, mentioned a case of Dr. Arthur's that came under his notice, where a superior bi-cuspid with only the outer half of the crown remaining, was built up so that it articulated perfectly with the lower one.

In regard to cylinders, he had used them for twelve years. Dr. Clark had said at Philadelphia, that he was the original discoverer, and he had no doubt he was *an* original discoverer.

Dr. CLARK, of New Orleans, said he borrowed the idea from Dr. F. H. Badger, supposing that he used them entirely. Dr. B. would not state how he did it, but he (Dr. C.) went to work to find out, and as he thought discovered it. He shortly afterwards found that Dr. Badger repudiated the idea entirely that teeth could be filled with cylinders alone, saying that he only used them in the centre of the cavity. He did not claim any original discovery and never had. What he had learned, he gave freely to the profession, as it was every man's duty to do.

Dr. SEARLE did not himself claim any originality ; the idea came to him in 1840, through a student of Dr. Keep, of Boston.

Dr. HESSEL, of New York, had not yet seen any better work done with crystal gold than could be done by the same operators with foil. He was one of the earliest to experiment in the use of sponge gold. He manufactured it himself in the most pure manner by means of an electro-galvanic battery; but it was expensive and therefore impracticable. Sponge gold in a pure state was essentially the same as gold foil, and subject to the same conditions. Though it would not change when tested by strong acids, yet in some mouths it would turn black, showing that the fluids of the mouth was a powerful solvent. Gold foil, if rightly and skilfully used, would stand all the tests required.

Dr. McKellops, of St. Louis, had been very unfortunate at first in the use of crystal gold, whether from imperfect manipulation or because the article obtained was imperfect, he could not say. He found that the plugs would change after a lapse of time. The article first used, however, was condemned by Mr. Nichols, of the firm of A. J. Watts & Co., who furnished him with a better article, which so far as he had used it, he found excellent. For large, saucer-shaped cavities with a small margin to hold in the fillings, he had found it very advantageous. It should not be condemned after three or four trials. He had used some five or six ounces of the superior article, and he believed that by the next meeting of this Convention, they would all be satisfied with it. He was very much pleased however, with Dr. Arthur's method, and was going to Philadelphia to learn it.

Dr. Gunning, of New York, said that according to the general experience of gentlemen who had used crystal gold, it required a great amount of pressure to make it solid. The density of the filling was a matter of great importance, and it seemed that more labor and time was required upon crystal gold than upon foil. That being the case, though a strong patient of forty years of age might well bear the great amount of pressure requisite with impunity, it would be frightful, perhaps, to a young delicate female. Time, to a person suffering pain, is a matter of some consideration. There were certain fillings which on account of their not being subject to wear, did not require so great density as others; would it be proper in such cases, under an excited condition of the nervous sensibility, to put in a larger quantity of crystal gold, subjecting the patient to great suffering, and running the risk in some cases, perhaps, of ruining the tooth in the socket, when foil could be inserted in less time and with less pressure and pain. The object of the dentist should be, not to see how dense a filling he could make in all cases, but to make a filling which would in all probability outlast the tooth in the socket. He contended that theoretical nicety was not their aim, but to make fillings best adapted to the particular cases. He would not concede that crystal gold was the best article in cases which would not admit of great pressure; and in gold foil, he insisted, that they had an article which they could control, and with it they could, in most cases, fill cavities in such a manner as to perfectly exclude moisture and save the teeth. The foil manufactured was not all equally adhesive, but they could use the more adhesive foil for cavities where the greatest care and nicety was required.

Dr. G. not having completed his remarks, it was moved that the speaker be allowed ten minutes longer.

Dr. BALLARD moved as an amendment, that the discussion continue for the remainder of the morning session.

Dr. CLARK, of Louisiana, opposed the motion, as he wished some time to be devoted to the exhibition of improvements in instruments.

Dr. TAFT moved an amendment to the amendment, by continuing the discussion till one o'clock.

Dr. SEARLE said there were complaints that the old hackneyed subjects were kept before the Convention, and day after to-morrow would be Sunday.

Dr. RICH moved the previous question, which was carried.

The question being put on the amendments severally, and on the original motion, they were all rejected.

Eight minutes being left of the time allotted to this discussion,

Dr. FLAGG, of Philadelphia, obtained the floor, and remarked that, as regards the materials for filling, they knew infinitely more of foil than any other preparation. Work done by such men as Hudson, and many other deceased brethren of the profession, had stood forty, fifty, and sixty years, and fillings made twenty-five years ago, could not be told from work done twenty-four hours ago.

As to the method of using foil, it should be so used as to be the most thoroughly condensed, with the least amount of labor. He had been pleased with the remarks of Dr. Arthur.

Dr. TAFT moved that this subject be resumed at four o'clock this afternoon. Lost.

THE FEES QUESTION.

Dr. Taylor from the Special Committee, to whom was referred the paper of Dr. Townsend on "Professional Fees," reported that they regarded the subject as one of great importance to the profession, and the views therein expressed, as embodying principles which alone can lead to professional excellence; and while we would thus commend the whole, we would especially draw the attention of this Convention to that part which treats upon professional counsel and advice. The committee would not pretend to even suggest the amount of compensation which should be charged for any given operation, yet we cannot but recommend that this Convention do express their decided disapprobtion to all that course of conduct, which so cheapens dental operations, that the good of the patient must be the sacrifice. We entirely disapprove the idea that an intelligent people will not appreciate and pay for the most perfect operations. We offer, therefore, the following resolutions:—

*Resolved*, That it is the duty of every member of the profession to so charge for his services, that he shall be well paid for all the time and the best skill he can expend on an operation, and which shall be an inducement for further excellence.

*Resolved*, That the best interests of dental science demand that a fair and liberal fee shall be charged for professional counsel and advice.

*Resolved*, That our profession, having for its basis true knowledge and skill, we cannot but regard that knowledge which may prevent disease as of equal, yea, more value to our patients than that which may arrest or cure.

The report was adopted unanimously.

Dr. Rich moved that a committee of three be appointed to revise and prepare the report of the proceedings for publication, and to have the revised report and Dr. Townsend's paper published entire, in all the dental journals. Adopted. The Chair appointed Drs. Rich, Maguire, and Dwinelle as such committee.

Dr. Van Patten, of Washington, D. C., moved that at five o'clock this evening, the Convention proceed to consider the time and place of the next meeting. Carried.

A notice was then read, inviting the dentists from abroad, to an entertainment, to be given by the dentists of New York and Brooklyn, at Dodworth's Rooms, this evening. The members to meet at Hope Chapel, at eight o clock, P. M.

Dr. Clark, of Louisiana, moved that one hour be now devoted to the exhibition of Dental Improvements.

Dr. W. B. Roberts, of New York, exhibited a set of teeth made with continuous gum mounted on platinum.

Dr. Loomis, of Cambridge, Mass., presented a specimen of artificial teeth for which he claimed superiority, dispensing with a metallic plate, and substituting a mineral base, the whole forming a solid piece.

Dr. Wheat, of New Haven, produced a specimen of teeth inserted in hard rubber compound. The hard rubber was perfectly free from any liability to absorption, and it was impossible to break the teeth so inserted, especially the grinders.

Dr. Mallette, of New Haven, presented another specimen, made in a similar way, which he and his partner had, he believed, perfected. There was nothing but mineral teeth and hard rubber used—no metal. He proceeded to describe the method of preparation.

From all that could be gathered by the reporter, it is believed that there is a patent which bars the free use of each of the above improvements ; in the two latter cases, however, it is only the use of Goodyear's Vulcanizing Patent that stands in the way.

Dr. Franklin, of Newark, exhibited a fluid lamp adjusted on a balance, with an inverted syphon, running from the cup to the wick. It was so adjusted, that as the fluid became exhausted, the part containing the wick gradually lowered, causing a uniform flow of alcohol. It had also the advantage from the arrangement of the syphon, of being perfectly safe from explosion. When the lamp is not in use, and the cap is put on the wick, then, the wick part being the heaviest, it was kept in a horizontal position by a spring underneath.

Dr. Malette exhibited a plate punch so arranged that two holes could be punched at once, corresponding with the pins for the backs in artificial teeth, one punch being moveable. It was used for punching two holes in the plates, suited to the pins in the artificial teeth.

Dr. HARRIS, of Baltimore, exhibited an instrument invented by Dr. Putnam, for producing local anasthesia, very useful for extracting teeth without pain.

Dr. PUTNAM stated that the agent used was ice and salt, and the instrument was so contrived, that the application could be made to the smallest portion of any external part of the body. The gums were frozen by the application, and consequently the teeth were extracted without pain. Some gentlemen raised an objection to this application, on account of its causing sloughing sores in the gums.

[The reporter here feels it his duty to state, that the report which appeared in the *Express*, concerning this invention, in which it is stated that the Convention adjourned to Dr. Putnam's house to witness the operation, is incorrect, and the fact of the report appears under the head of "Afternoon Session," when the explanation was made in the morning, leads to a suspicion that the reporter had some other purpose in view, than to give a true and fair account of the matter, and the subsequent use made of that report confirms the suspicion.]

Dr. TAFT, of Ohio, exhibited a blow-pipe for throwing a warm jet of air into cavities for the purpose of drying them. It consisted in an india-rubber bag, with a metal tube attached, which might be filled with a heated substance that would retain heat well, the air passing through it by pressing on the bag.

There was also exhibited an instrument to enable dentists to get a more perfect articulation of teeth.

The Convention took a recess till 4 o'clock.

### AFTERNOON SESSION.

A motion was made and carried, that the Convention resume the consideration of the subject of the best preparation of gold for filling teeth.

Dr. McQUILLEN said that his experience had not been a successful one in the use of sponge gold. But gentlemen would say that his experience had been so limited, that he could not arrive at correct conclusions in regard to this matter. He had not arrived at the conclusions he had stated, so much from his own experience, as from the failures of eminent operators. Therefore he stood forward as a witness in favor of the objections urged against sponge gold, that its use produced discoloration and disintegration of the teeth.

He had tried the plan of annealing, explained by Dr. Arthur, and found that he could not introduce as much gold into a given cavity, as with the ordinary gold foil that he received from Abby. He believed the annealed gold hardened under the instrument so rapidly as to choke up. There was a point where they must cease to use the instrument when operating with ordinary gold foil, as there was a point where the painter must lay aside his pencil ; otherwise they might get such a temper in the gold that the next gold put in would not adhere. He had reason to infer from his experience in the use of annealed gold, that the specific gravity of a filling, was not equal to that of one made with the ordinary foil, judging from the quantity used in the cavity.

Dr. Ballard said that he could speak with some confidence upon this subject, gained by an experience of some years. He had used crystalline gold for three years with great success. He had the pleasure of seeing the first operation that was ever performed with crystalline gold, and it was perfectly successful. The result of his experience could be summed up in a very few words. There was no question that a vast amount of improperly prepared gold had been in the market, imperfectly purified and imperfect in its microscopic structure. He wished only to speak of perfectly made gold, which contained all the requisites that were desirable for a successful operation. Many failures, undoubtedly, had occurred with the very best gold, but his own experience taught him that these failures had been the result of imperfect manipulation. He did not know a man anywhere who did not make a failure sometimes. In a majority of cases occurring in his practice of three years, he had used crystalline gold with success, but there were cases in which it would not answer. He wished to state the following reasons in favor of its use—its exceedingly delicate structure, which enabled the dentist to place it in positions so exposed, that nothing else could be retained there, its perfect purity, and its density.

In regard to porosity of fillings, it was perfectly evident that a perfectly solid filling could not become porous without expanding and disrupting the plug, or splitting the tooth. A porous filling, therefore, must have been left so in the beginning ; gold once deprived of its porosity, could not become porous again. The experiment related yesterday, by Dr. Dwinelle, ought to convince any one that a filling of crystal gold could be made perfectly solid.

Dr. TAFT considered the method adopted by Dr. Artnur, as a very great advance in the use of gold, and he intended to try it. Leaving that out of view, he preferred in most cases crystal gold to gold foil. There was a confusion of terms in speaking of sponge gold, many applying it to all the preparations that had been made and called by that name, perceiving no difference between the varieties that had been produced. There were three forms in use for filling. Sponge gold he considered to be simply granulated gold obtained from precipitation ; by various methods of precipitation they could get an article with which they could fill deep cavities. Then there was structural or fibrous gold. Then again there was another form in which its crystals were larger and more definitely formed, than in the two previously mentioned. In that variety which had no structural character, but was simply gold in a state of minute division, they had to depend entirely upon its property of cohesion in introducing it into a cavity. This form of sponge gold was not reliable, although occasionally tolerable fillings might be made with it. In the structural or fibrous gold—which might be denominated crystalline—besides the adhesiveness, it was retained in the cavity by the fibers folding upon one another. Again in gold formed of definite crystals, they not only had the cohesion property, but the interlacing of the angles of the crystals to retain the filling in a solid state. In the use of foil well annealed, there was a cohesion doubtless sufficient to retain the particles together ; but in the crystalline gold there was besides cohesion, this interlacing of the particles which they did not have in foil. No doubt with sufficiently strong walls they could build up foil into a pyramid as described by Dr. Townsend, but he thought it required much more skill with foil than with crystal gold ; and he conceived that there were many cases where crystalline gold could be used where foil could not, as in the case described by Dr. Clark, where one-third of the approximate edges of the incisors are broken away.

The PRESIDENT (Dr. Harris,) said that the preservation of the natural teeth was of more importance than the replacing of those organs by artificial substitutes. He rose not so much to give his own experience in the use of crystalline gold, as the results he had seen from its use by other operators. He had been in the habit of using crystal gold for three or four years. During the first two years of his practice in using it, the results were not so satisfactory as he could have wished, but more recently they had proved so, although he was not yet prepared to lay aside the use of foil. He used three ounces of foil to one of crystalline gold. When he had heard doubts expressed by several members of this Convention, with regard to the practicability of making fillings with crystalline gold of as much value and permanency as those made with foil, he felt it due to the manufacturers of crystalline gold to say, that he had seen fillings of this material that had been used in the mouth upward of two years, which were in a most perfect state of preservation, equal, and in some cases, when all the circumstances connected with the cases were considered, superior to any fillings with foil.

A case occurred to him at this moment, of a young lady who formerly resided in this city, who recently came into his office to have her teeth examined. Many of her teeth were made up almost apparently of gold, several crowns having been destroyed. One of these crowns, in particular, had been built up with crystalline gold, and although it had been there two years, it was as perfect as any filling could possibly be. There were thirty fillings or more in all, made by Dr. Ballard, of crystalline gold, two years ago. An upper molar that had been decayed away, so that if he was not mistaken, the walls were entirely gone with the exception of a small portion which came down below the margin of the gum, was built up and answered all the purposes of a natural tooth. He could name other operators, Dr. Dwinelle particularly, who had realized his fullest expectations with regard to this article. He was fully satisfied that there were cases in which this form of gold could be used more advantageously than foil; but on the other hand, he might say the the same in favor of foil. It would be difficult for him to say which he regarded as the most valuable, though if he could have but one he would hold on to the foil, having used it so long that he had become in that respect, something of an "old fogy."

Dr. Rich said, the difficulty of procuring foil that was sufficiently adhesive, even after he had annealed it, had induced him to try the merits of crystal gold. The experiments he had made with it, had demonstrated beyond a doubt, that its particles would adhere, it could be made solid, and when solid it was impermeable.

Among the experiments made to ascertain these facts, were the following: Portions of this gold were packed in the cavities of teeth with ordinary instruments that he used every day in his practice. One of the filling so formed was drawn out into wire, another was rolled into plate, and a third was hammered into plate on the anvil. Another portion that formed a small disc about an eighth of an inch thick, was secured on the end of the tube of an air pump; a drop of water was then placed upon the upper surface of the disc, and the tube exhausted with the full force of the pump, (which was a powerful one,) the water remained upon the surface. The disc was then ground down to about one half its original thickness, and the experiment repeated with the same result. The objection to this gold that it requires more time to consolidate it than is necessary for foil, will not be sustained when it becomes better known. When he first used it, a filling of crystal gold required double the time that he would have spent on one of foil. Now he packed and finished it with as great facility as he did foil, and found it more easy to prepare, of convenient sizes, for introducing into the cavity. In difficult cases, as, for instance, where the filling has to be built up independent of the support of the walls of the cavity, crystal gold can be used with much greater facility than foil. This, is a very important advantage. The improved crystal gold, as manufactured now, by A. J. Watts & Co., did not of itself produce discoloration, it was *pure* gold without the least trace of any other substance, and therefore it could not discolor the teeth.

Several professional friends had told him, that they had cases in their practice, where they had used crystal gold, and it had produced discoloration ; as he (Dr. R.) doubted that the gold had been the cause of that effect, he had requested them, for their mutual satisfaction, to allow him to see the fillings removed, and to examine the cases critically. In several instances they had done so ; the fillings were removed, and the gold, and the cavities examined, and in every case it was clearly evident that the fault was not in the gold, but in the manipulation, either in preparing the cavity or in packing the gold, and in every case, it was easy to decide to which of these two causes the failure was to be attributed.

The statement is often made, that the surface part of a crystal gold filling, becomes quite solid and hard, while the rest of it remains soft and porous. When this occurs, it is the fault of the manipulation ; if one part of the filling could be made solid, the whole could. The amount of pressure that made the surface dense would have had the same effect upon any other part of the filling, if it had been applied there. The proper method was, to pack the filling solid from the bottom of the cavity, and introduce the gold in small portions, each of which must be made as solid as may be desired, before the portion which is to be packed on top of it is introduced. One of the most valuable properties of crystal gold is, that it can be made of any given degree of density of which gold is susceptible, with much less pressure, than would be necessary to produce the same degree of density in foil. In finishing the surface of the gold, when the cavity is filled, this peculiarity must be borne in mind, and in consolidating and finishing the gold at the margin, great care is necessary to avoid working it too much at that point, for if as much labor was spent upon it, as would be necessary to make the margin of a filling of foil as solid and as hard as it ought to be, the margin of the crystal gold filling would become brittle, and would easily break and crumble up. The same effect would be produced with foil ; when it reached the same degree of density, that would also become brittle.

PLACE OF NEXT MEETING.

The Convention then proceeded to appoint the place for the next meeting.

Washington, Boston, Niagara Falls, Saratoga, White Sulphur Springs, (Va.,) Baltimore, Cincinnati and St. Louis, having been severally proposed, the Convention decided, by a division of 56 to 40, that the next meeting shall be held in Boston.

On motion, it was agreed that when the Convention adjourn, they adjourn to meet at Boston, on the first Tuesday in August, 1857.

LOCAL SOCIETIES.

Dr. Blandy, of Baltimore, offered the following resolution, which was adopted:—

*Resolved,* That this Convention recommend the formation of local or state societies, and that each association thus formed, be requested to send one or more delegates to each meeting of this Convention, thus making our annual convocation emphatically the great central Congress of the Dental profession.

CREDIT FOR NEW IMPROVEMENTS.

Dr. Taylor moved the following resolution, which was adopted:—

*Resolved,* That in the opinion of this body, the credit due for new discoveries or useful modes of operating, belongs more to those who have given those improvements to the profession, than to those who pretend to have discovered the same at a previous period.

The following resolution was offered by Dr. Buckingham, and adopted:—

*Resolved,* That the Corresponding Secretary request the members of this Convention and others, who have anything new or useful, to present them at the next meeting.

SCALE OF PRICES.

Dr. Shaw, of Philadelphia, moved the following resolution:

*Resolved,* That a committee of be appointed to inquire into the expediency of adopting a scale of minimum prices, every practioner to have the privilege to have a scale of prices of his own, as much higher as he may consider proper, and that it shall be considered derogatory to the character of any dentist, to go below the scale that may be adopted by the Convention; said committee to report at the next meeting of the Convention.

The resolution was adopted, and the Chair appointed as the committee, Drs. A. R. Shaw, C. W. Ballard, and E. Townsend.

### COMMITTEE ON PUBLICATION.

The following resolution, offered by Dr. Bonsall, was adopted :—

*Resolved*, That the committee on the publication of Dr. Townsend's address, be authorized to draw on the Treasurer for the expense of publishing the twelve hundred copies ordered by the Convention.

The following resolution, offered by Dr. Rich, was adopted :—

*Resolved*, That the publishing committee be authorized to draw on the treasurer for the expense of furnishing stereotype plates of the proceedings of the Convention, and Dr. Townsend's essay, to such of the Dental Journals, as shall publish them entire.

### VOTES OF THANKS.

A unanimous vote of thanks was then passed, on motion of Dr. Austin, to the Dentists of New York, Brooklyn and Williamsburg, for the courtesies received by the members from abroad, and also, on motion of Dr. Watt, to Messrs. Jones, White & McCurdy, for their excellent entertainment on Thursday evening.

A vote of thanks was then, on motion of Dr. Coates, of Virginia, tendered to the worthy President of the Convention, for the dignity and impartial manner with which he had presided over the deliberations.

The Convention then on motion, adjourned *sine die.*

### THE COLLATION AT DODWORTH'S.

On Friday evening, the Dentists from various portions of the United States, who have been in attendance at the Dental Convention, were invited to attend a collation given by the profession of New York and Brooklyn, at Dodworth's Hall, near Grace Church. The tables were laid out in the usual manner of such entertainments. They were tastefully covered with fruits, ornamental dishes, etc.

The following were the Committee of Arrangements :—Drs. J. H. Foster, E. J. Dunning, J. G. Ambler, J. Allen, C. M. Ballard, N. W. Kingsley, C. C. Allen, W. H. Dwinelle, F. H. Clark, B. F. Lord, W. T. Larouche and B. C. Leffler.

Some two hundred and fifty of the Dental profession took their seats about nine o'clock, and commenced a systematic attack upon the good things before them.

Dodworth's band enlivened the entertainment by the performance of exquisite music.

Previous to the commencement of the repast, Dr. Foster, Chairman of the Committee of Arrangements, addressed the guests as follows :—

Mr. President and gentlemen of the American Dental Convention—

A portion of the Dental profession of this and the adjacent city of Brooklyn, have invited you to this entertainment to-night, and have assigned to me the pleasant office of expressing to you in their behalf, the satisfaction they feel in the opportunity thus afforded them, by your meeting in this city. In their name, and in their behalf, I bid you a cordial welcome.

I behold around me representatives of our profession from many different, and some distant States of the Union.

Our country has expanded to such a degree, that we cannot expect every State to be represented in a local Convention like this. But from the Green Mountains of Vermont, and the Granite Hills of New Hampshire in the North, to the everglades of the warm and sunny South---from the far and distant West, "where the star of Empire takes its way," and the wilderness has been made to blossom as the rose, to this rude and rock-bound Atlantic coast—throughout the whole length and breadth of this vast extent of territory—wherever art and science have extended their onward march—there may be found those who have chosen this profession as the occupation of their lives.

You have here, gentlemen, not in point of number only, but in intellectual and scientific attainment, a most honorable and respectable representation of the profession; and I most sin cerely wish that many others whom we all know and delight to honor for their professional worth and usefulness, were here also to-night, that we might extend to them also, the sincere and hearty welcome with which we greet you. (Applause.)

At the principal table were seated on each side of Dr. Foster, Dr. Harris, of Baltimore, the President of the Convention; Dr. Rich, of New York, the ex-President; Professors Townsend, Arthur, Buckingham and Flagg, of Philadelphia; Professors Taylor, Taft and Watt, of Cincinnati; Professor Austin, of Baltimore; Dr. Neall, of Philadelphia; Dr. Clark, of New Orleans, and among the invited guests, we noticed Mr. Ashael Jones, S. S. White, and J. D. McCurdy, of the firm of Jones, White & McCurdy.

After the edibles had been abundantly discussed, Dr. Foster again addressed the company, congratulating them upon having just closed one of the most happy Conventions that it had ever been his pleasure to have known. He regretted that indisposition had prevented him, in a great measure, from being present at the deliberations which had been attended with so great unanimity of feeling, and hoped the future meetings would be attended with like results. They had learned how much had been done to advance the interests of the Dental profession, and how much remained to be discovered. Their lives were fleeting but their art was not;

> "And though our hearts are strong and brave,
> Still like muffled drums they're beating
> Funeral marches to the grave."

Dr. Neall responded to the remarks of Dr. Foster. He said he rose because he was called on, and always liked to answer when he was called. He would have much preferred however, that their great Goliah of Gath had *Harrassed* them, (laughter) a man whom, if he had not performed his part so well, he should have considered of entirely too large make for a dentist—and he told him long ago, that a smaller man could work much better around the chair, than one so large as he.

As to that funeral march just alluded to, all he had to say was, that he presumed he expressed the sentiment of all present, when he hoped it would be a very slow one. To the other sentiments expressed by the Chairman, he heartily responded, and in behalf of the members present he would say, that the welcome tendered had been accepted—they had made themselves at home already. (Applause.)

The Chairman at the opening had said, that the star of empire westward took its way. There were here present, brethren from west of the Alleganies, who had shown an evident determination on their part, that that star should never set, until the last ray of science was exhausted. He was reminded of the words of a Western poet,—

'By mountain height, in lowly vale,
By mighty lake and gentle river;
Wherever sweeps the chainless gale,
Onward still they speed forever."

Such, he trusted, would be the march of the dental science. (Applause.) But while their march was onward, he hoped it would be borne in mind that the great end of their science was to do good. He was pleased with the remark made this afternoon, in favor of holding the next convention at Boston, because they could do more good there. The highest and noblest ambition was to do good—to succour human woes. (Applause.)

THE REGULAR TOASTS.

The first regular toast was—

The President, the Constitution, and the General Government of the United States of America.

Music—Hail Columbia.

The second regular toast was—

The American Dental Convention, organized upon the broad basis of equality and fraternity; may it prove a foundation for the erection of a superstructure, beautiful and harmonious in all its proportions, the materials of which shall ensure its perpetuity.

To this Professor HARRIS, of Baltimore, responded. He remarked, that he had witnessed in this gathering of Dentists, a scene he had never expected to behold, and after referring to the rapid progress of the dental profession, he compared dentistry thirty years ago, to a wilderness overgrown with wild forest trees; since then it had been nearly cleared, still *roots* remained to be extracted. He thought, however, from what he had seen this evening, they all understood the operation of "*filling*" *well.* (Laughter and applause.)

Third regular toast:

The originator of this Association, who, residing in the city of brotherly love, has infused and incorporated that spirit into the whole body politic.

Dr. Townsend, of Philadelphia, was called upon to reply, and was greeted with warm applause. He felt proud to see so large an assembly of intelligent, well educated gentlemen present, and to know that his individual efforts had contributed to bring this result about. One year ago, when he first spoke of the possibility of collecting the dentists of this country, and possibly of the world, into a Convention, upon a broad democratic platform, and thus eminating a bond of union, the thing was doubted by his best friends. It had been a pet scheme of his for years, and he could not relinquish in spite of those discouraging words; accordingly, in the spring of 1855, he took the liberty of calling his friends together, and laying the scheme before them. In this, he had followed the custom of the Quakers, among whom he was brought up, who when they have anything for a long time resting upon their minds, lay it before their brethren, and if their friends are ready to receive it well, if not they wait till they are ready to act with them. He found his friends willing to act, and now he felt that the bond of union was secured, and that the next Convention would be even larger than this. (Applause.) So long as they kept to the fundamental principles of truth, love, and charity, they would gain in numbers, and promote the great end of doing good. (Applause.)

Fourth regular toast—

The first President of the American Dental Convention.

Dr. Rich responded as follows:—Mr. President and Gentlemen. The success of the American Dental Convention has made it a great honor to have been its first president. The scheme, of a National Convention of Dentists, is no longer a doubtful experiment. It is a successful one. And the result is the establishment upon a firm basis, of a useful and permanent institution, which, if we love our profession, we must all be proud of. Associated effort for the accomplishment of any purpose, is one of the great engines of civilization ; and the talent and general intelligence of the Dentists of this country, has rendered an association like this, an absolute necessity. When a number of ambitious and well educated men, situated in different localities, of a large country like this, are earnestly striving to elevate and advance the standard of their profession, they need some common ground, such as this society presents, where they can come together, and contribute the result of their individual labor, to the general fund of information. And the beneficial effects of associated effort, for the advancement of the cause of science, is nowhere more apparent than in the history of our profession. It was only when Dentists began to communicate freely with one another on professional subjects, and thereby elevate themselves, that our calling began to be recognized as a respectable one.

It is scarcely twenty-five years since the first efforts were made in this direction. The few liberal-minded men who started this movement, saw the necessity of united effort, and such of them as resided in this state, formed a local Dental Society, in the city of New York. A few years afterwards, the Baltimore College of Dental Surgery, and the American Journal of Dental Sciences, were established. And then in 1840, came the grand movement in the history of our profession. A few of the distinguished dentists of our country, I believe they did not exceed twenty-five in number, met in this city, and formed the American Society of Dental Surgeons. From that moment our profession has been steadily advancing, and now a call for a National Convention, is responded to by nearly two hundred members. The pioneer journal, whose first issue was barely one hundred and fifty copies, has reached an issue of over a thousand, and there are five or six other Dental Journals, with a circulation of from one to three thousand copies each. We have three or four Dental Colleges in successful operation, who swell our ranks every year with earnest, well-educated men. (Applause.) A number of local societies have been established, in different parts of the country; these organizations have stimulated and benefitted all who have been brought within their influence. Gentlemen, such is the condition of our profession in this country. We see what the Dentists themselves have done. Is the credit of the progress and elevation of this profession due to them alone? Why is it that the Dentists of other countries have not elevated themselves and their profession? And to what cause must we attribute the superior skill and intelligence of the American Dentists, when compared with those of other countries? The reason is obvious; the patrons of the European Dentists are the privileged classes, the people of rank, the rich, and the few, a very small portion of the population. There, where a small number live in luxury and plenty, at the expense of the poverty and privation of the many, the down trodden masses have no means to pay for dental operations; they cannot procure the necessaries of life, much less pay for the services of a skilful dentist. The Dental profession is at a low standard. They have no elevating influences. No Dental periodical literature. Dental Colleges have not been attempted. Dental Societies they have never had, and consequently, they produce no men of superior talent; and we see a comparatively young nation, supplying the Old World, with one of the means of refinement; for America, actually, furnishes Europe with Dentists. (Applause.) How different a condition of things do we find here. The high position our profession occupies, is only one of the types of our beloved country. Its condition is one of the results of the working of the problem of self government by the masses, and we flourish, because we live among a highly prosperous, well educated, moral, and refined people, the masses of whom can appreciate our skill, and afford the means to patronise us. None but the refined, pay the necessary attention to the teeth, and in this respect, and that of personal cleanliness, the people of this country are elevated far above any people on the earth. (Great applause.) Gentlemen, how many things we have to be proud of as Dentists. In this soil, so genial to the growth of talent, our profession has advanced with giant strides. We have the only periodical literature of Dental science in the world. The only regularly organized colleges, for Dental education. The only Dental Societies, and the only Dental Convention that has ever been held in the world. (Applause.) One of the great advantages of this Convention is, that it has brought a large number of the members of our profession face to face. We have seen the men of whom we have heard. As a general thing, men needed but to know one another, to like one another, and when brought together, to converse on topics of mutual interest and concern, they generally became good friends. And, one of the results of which we ought especially to be proud, is that, instances have occurred during this meeting, of gentlemen, who on account of some old rankling animosity or jealousy, have not spoken together, or recognized one another for years, having come together at this Convention—taken each other by the hand—and all their former ill feeling had been swept away. (Long and loud applause.) Another of the fruits of this Convention is, the New York Dental Association, which was organized on the eve of the meeting of this body. I sincerely hope there may be many such formations throughout the Union, before the next meeting of this Convention. (Applause.)

Allow me gentlemen, to express to you my warm acknowledgments, for the honor of this toast; and, to thank you again, for your kind support, during the time I had the honor to preside over your deliberations. (Applause.)

Fifth regular toast—

Dental Colleges.—Ever keeping pace with the spirit of the age. May their motto be "Excelsior."

Professor ARTHUR, of Philadelphia, said he was rather surprised that he should be called upon to respond to this toast, being the youngest in the category of professors present, nevertheless, he had the honor of being the oldest graduate of any dental college. (Applause.) He well remembered the struggles of his respected friend, Professor Harris, at the outset of this enterprise, which had led to such important results already, and which would lead to more important hereafter. He regretted to say, that the opposition which was at first met, was not yet entirely broken down, though it was rapidly passing away. It must be evident to every intelligent dentist, that a regular systematic education was absolutely necessary for the advancement of the science, and so far as he was acquainted with the gentlemen connected with the Dental Colleges, they were honestly seeking that advancement. (Applause.)

Dr. A. then explained the reason of the sudden passing out of existence of the institution, with which he had been connected, and its being succeeded by another. It resulted from an attempt to bestow honors by the board of trustees of the Philadelphia College, only one of whom was practically acquainted with the dental art, upon individuals not known to the profession at large, independent of the wishes of the faculty of that college. This practice had grown up in literary colleges, established by endowments from educated men, without whose consent, or the consent of teachers employed by them, no degrees could be conferred. As they were intelligent men, they were capable of judging of literary qualifications, and therefore, it was proper enough that they should confer degrees. But it was easy to see that such a rule, when applied to a college erected for the purpose of professional instruction, must be destructive of its interests, and injurious to the profession upon which it was attempted to be practiced. The trustees of such an institution were, of course, totally unqualified to confer honors. Therefore, the gentlemen of the profession connected with that college, sacrificed its charter, and, though at first doubtful of success, they succeeded in obtaining a new one, containing a clause expressly prohibiting the conferring of honorary degrees, except by the consent of the faculty. That faculty intended to do their duty regardless of any other consideration, and if that was done, the course of the dental profession must be onward. (Applause.)

Sixth regular toast—

The Dentists of the South and Southwest.—A noble army of co-laborers. May they swell the tide of our progress with a ceaseless flow, like that of their own mighty Mississippi.

Dr. J. S. Clark, of New Orleans, responded.

He said that large rivers, large growth of tropical verdure, and genial skies, might not produce large men to "*swell the tide*" of human progress; but they engendered large aspirations. (Laughter and applause.) True to these impulses, they might attempt large things, but they felt that as in nature they possessed an artery of a Continent, so, professionally, they felt the throbbings of arterial blood, whose every pulsation was in unison with the great heart.

He would illustrate. A friend of his, being fond of oysters, called Tike his servant, and told him to polish his chafing dish and to make it shine like the sun. Tike took the tin and worked at it a long time, but he could not produce anything like the lustre of the sun. Tike hated to give it up, but finally came back to his master and said: "*Massa, I make im shine like de moon, but he no shine like the sun. Massa, I think he can't be did.*" (Laughter.) So in the dim twilight of our novitiate, high impulses may lead us to think ourselves creative geniuses; that we are called upon to even out-artist nature herself. But if we are true servants of our great master, when we find our mistake, we shall do as Tike did, keep on with our work, for Tike's work was not done. His master called him late one night, and told him to brush his boots, and to make them shine so that he could see his face in them. Tike took the boots, and by the dim light of an old candle commenced his task, but the more he brushed, the more he couldn't see his face in them. Faithfully he worked, till finally he began to see the reflection of an eye, then his mouth, and he exclaimed, "*I see um eye! I see de mouf! de face come bym-by!*" (Laughter.) But Tike did not know that a light had broken in from the east, and that the sun had risen on his toil-spent night.

We, gentlemen, have watched the sun in his rising. We saw at first the grey dawn, then a ray of light came down from Baltimore and the cities of the North, till a broadcast sunlight made us feel that ours, indeed, was the "Sunny South." (Applause.) But some of us love to come home. Some of us come on a yearly pilgrimage to the old hearthstone. We listen to the gentle footfall that rolls back on memory the tide of years, and we stand once more in boyhood's "home, sweet home." With this feeling fresh in our hearts, may we not visit this, our annual re-union, as the place where our friends and brethren dwell. (Applause.)

Seventh regular toast—

Our brothers of the great Northwest.—Full of vital energy, may they be true to their motto: "The Young Americas of our profession."

Dr. ALLPORT, of Chicago, in response said, that there was a familiar song whose chorus was, " There's a good time coming." That good time in the dental profession had come. (Applause.) It did him good to see the veterans of the profession here mingled with those who, like himself, were young, but were willing to shoulder the responsibility of elevating it to that dignity that its devotees expected it to attain. (Applause.) A good time had come, but a better time was coming, when dentistry would take its rank with the most respectable professions. What was there that was more needed than intelligent dental practitioners. The time was coming when every place that supported a physician, or a lawyer, would also support a well educated dentist. Their numbers here to-night were respectable, but nothing like what they would be (applause), and while they increased in numbers they should equally increase in scientific knowledge and skill. He assured them that the members from the west would return with the determination that the west should be the Young America of the profession. (Applause.)

Eighth regular toast—

Our brethren of the New England States.—" Stars in the East," their cheering, effulgent rays, gladden us here to-night.

Ninth regular toast—

Dental Progress.—May its march be onward and upward, until its genial rays shall be reflected throughout the world.

Dr. ALLEN, of New York, said that he recollected being present on one occasion, when Davy Crockett being called on to address an assembly, came forward, and, after standing a moment or two in a very awkward position, said : " My good friends and fellow-citizens :—There is not a man on earth that would love to talk to you better than I, if I only knew how." (Laughter and applause.) I feel like endorsing that sentiment this evening.

Dr. A. then spoke of the history of the progress of the dental art, remarking that more progress had been made in the last half century, than in the previous thousand years, and American genius had done more in that time, than the genius of all the rest of the world. He also made some further remarks upon the mechanical department of dentistry, to which he was more partcularly devoted, urging the necessity of a thorough knowledge of the anatomical structure of the muscles of the face, in order to insure complete success in restoring the natural expression of the countenance.

Tenth regular toast—

The Ladies.—Often present at our labors, never absent from our thoughts.

Dr. Austin was called out to respond to this toast, and made some very amusing remarks which he termed an apology for not responding, and which he modestly requested the reporter not to give. His remarks created considerable merriment.

Dr. Gunning, of New York, being called for, rose and remarked that he could appreciate the feelings of his friend from Baltimore, living as he did in a city renowned for the beauty of its ladies.

Eleventh regular toast—

The Press.—Ever ready to aid in every good word and work, we invoke their especial help in this our new fraternal enterprise.

Mr. Burr, of New York, being called upon to respond, said that he considered it a work of supererogation to speak for the press, for every body knew that the press spoke for itself. Nor could any one, strictly speaking, be said to represent the press, because the press represented itself, however much it might *mis*represent others. Speaking for himself, individually, he would say that he had a very high appreciation of the dental art, and any gentleman, possessed of the general intelligence requisite for his station as a member of the press, who failed to appreciate that art, was unfit for his position. But though, paradoxical as it might appear, he was in a certain sense a speech-maker, yet being no speaker, he would resume his seat. (App'lse.)

The regular toasts being completed, the Chairman read a letter from Mr. Jones, accompanying a box of honey for the table, and remarked that having had the honey, he hoped they would in addition now have some honied words from Mr. Jones. Mr. Jones begged to be excused, but would call upon his partner to answer for him.

Mr. J. D. McCurdy, in answer to this call, related an anecdote of the man that Dr. Franklin observed at a public table. While every one was busy helping himself, this man, unlike the rest of them, said nothing, whereupon the Dr. was disposed to attribute to him some very good sense, and he watched him with a good deal of care, with the expectation that when he did open his mouth, he would hear something very brilliant. By and by the dumplings came on, and the man was desirous of having some sauce upon his dumpling. The Dr. eyed him pretty closely to see if he was going to speak, when at length he exclaimed to his neighbor : "Will you hand me some of that stuff you wallop your dumplings in?" (Laughter.) So possibly he (Dr. McC.) might have got the credit of being capable of making a speech if he had not been exposed. He, however, had sufficient notice at the beginning of the entertainment that it was expected of him, from the fact that the committee had brought him forward to a particular place, with a number of other lambs, and older sheep for slaughter. (Laughter.) When he was seated in his place he felt himself in the condition of the fellow who had been arrested for misdemeanor, whose counsel made him out quite innocent and reputable, so much so that he exclaimed that he never knew he was so good a fellow before. (Laughter.)

Dr. McC. then spoke of his fears at first for the success of this enterprise, on account of the many jealousies that existed among the members of the profession, but he confessed himself surprised and gratified at the result, and wished them continued success. (Applause.)

The following toast having been handed to the Chairman was then read :

The first president of the New York State Society of Dental Surgeons, to which

Dr. Covell responded. He said that he had frequently heard warm expressions of the great and lasting advantages derived from that institution, showing that though dead it yet speaks. (Applause.) It did not die for want of vitality in its members, but only of its organization.

After some further remarks, in which he alluded to the press as the mighty engine by which the dental science was achieving its progress, the gentleman sat down.

This allusion to the press called the remark from Dr. Harris, that no profession used the *press* more than the dental.

Mr. Bigelow, of the *N. Y. Express*, hoped to be excused in this connection, for giving the following sentiment:

Mr. Chairman and members of the dental profession:—When your profession next hold a meeting like this in our city, may all our distant and neighboring *Towns-send* still larger delegations to your Convention, where may the dental science be *Foster*-ed until all its *Rich* treasures shall be discovered, and fill both patient's teeth and practitioners' pockets. (Applause.)

Dr. Dwinelle, of New York, being called out said, that the star of hope was in the ascendent, the good time had come. How many unsuccessful efforts in the last twenty years had been made to fraternize the members of this profession, but for the last two years their efforts had run parallel with their wishes. The secret of their success was the democratic principle upon which they were organized; hitherto the efforts were made in a wrong direction, but now they harmonized with the spirit of their institutions. He hoped the designs of the excellent founder of this institution would be carried out to the letter, and that all unnecessary machinery would be avoided; and next year he hoped to see, not only American, but European dentists represented in this Convention. (Applause.) The name of a dentist, once a reproach, now respectable, would yet become a title of honor. Then the names of Hudson and Gardette (peace to their memory!) would shine forth in greater brilliancy; while he hoped the names of Harris, Townsend, Arthur, Dunning, and others, would by no means be forgotten. (Applause.) If every man would be true to the estimate that he put upon himself, the world would sooner or later endorse it. There was much to encourage them surrounded by circumstances like the present.

"Let us then be up and doing,
With a heart for any fate—
Still achieving, still pursuing,
Learn to labor and to wait."

(Applause.)

Dr. Gunning rose to give a sentiment, "May we never forget the Dental writers of Europe." He regretted the lack of eloquence to do justice to the subject, but he did not wish to see anything that would look like forgetfulness of those noble writers —Bell, Hunter, Fox, Toombes—therefore he deemed it his duty to draw attention to those names.

Dr. HARRIS was glad of the mention of those names, and would give as an additional sentiment, the memory of a noble pioneer who fell a martyr to dental science—The memory of Alexander Naysmith.

Dr. DUNNING, of New York, at the invitation of the Chairman, remarked that it gave him heartfelt pleasure to be present. Thus far he had been a man of action and of labor, and had never learned to make speeches. He alluded to his first connection with a Dental association, when they met in a small room. He was then full of enthusiasm, and hoped he had not entirely lost it now. He attributed the rapid growth and success of the profession, to the fact that it was not trammeled, as other professions were, by laws and edicts that had their origin in by-gone times. Each man was putting forth all his individual efforts to do his duty and contribute to the advancement of the cause. It was the motive, aim and aspiration with which the Dental practitioner performed his duty, that had given to this profession its present character. (Applause.)

Dr. TOWNSEND gave as a toast—The New York Dentists, whose generosity is only equalled by their devotion to science—May they ever remain the brightest jewels in the coronet of their States greatness.

Dr. BALLARD being called out said, that nothing but kindness of heart could have induced them to call upon him to respond, and nothing but that, stood between him and the well laid charge of presumption. Young America had been spoken of this evening, and so far as the younger members of the Dental profession were concerned, he claimed to be an humble representative. If he had any reputation in his profession, it was due to the fact that he entered in at the straight gate, and there (pointing to Dr. Harris,) sat the gentleman who made the path smooth. (Applause.) When it was proposed at Philadelphia, to hold the next Convention at New York, it was urged as a reason that there was no local society there, no union among the Dentists, and that therefore good might grow out of it. Good had grown out of it, for there was now union among them, and a New York Dental Association. (Applause.)

Dr. DWINELLE gave as a toast—

Our excellent representatives abroad. "Though lost to sight to memory dear."

The CHAIRMAN apologized for omitting, in consequence of the great number of the regular toasts, to call out their worthy friend, Dr. Taylor, of Cincinnati; he hoped to hear a few parting words from that gentleman.

Dr. TAYLOR said he had hoped to be allowed to sit in silence this evening. Unfortunately he had to make an apology instead of a speech for the close, as he had made no sort of preparation, and even if he had, it would have done no good. He began to think that the eastern people were "some," and next year he intended to go down to Boston and see what the real genuine Yankees were. (Laughter.) The dental science was once spoken of as auxiliary to the medical profession; that was now reversed, and the medical profession was auxiliary to that of the dental surgeon, and having got the upper hand, he hoped they would keep it. (Applause.) He returned his sincere thanks to the Dentists of New York for this hospitable entertainment, and hoped the gentlemen of the west would have an opportunity before many years to show their hands. (Applause.)

The festivities here closed, and the members dispersed.

